# Supplementary material for: Flexible control of vocal timing in Carollia perspicillata bats enables escape from acoustic interference
Source: Commun Biol. 2023 Nov 13;6:1153. doi: 10.1038/s42003-023-05507-5 (PMC10643407; doi:10.1038/s42003-023-05507-5)
Supplement: Supplementary file 1 — Supplementary Materials [file 42003_2023_5507_MOESM1_ESM.pdf]

Supplementary Materials: Flexible control of vocal timing in *Carollia perspicillata* bats enables escape from acoustic interference

Ava Kiai[1]*, Jan Clemens[2], Manfred Kössl[1], David Poeppel[3], Julio Hechavarría[1]*

Corresponding authors: ava.kiai@stud.uni-frankfurt.de, hechavarria@bio.uni-frankfurt.de

The file includes:

Figs. S1 to S8

Tables S1 to S17

Supplementary Figures

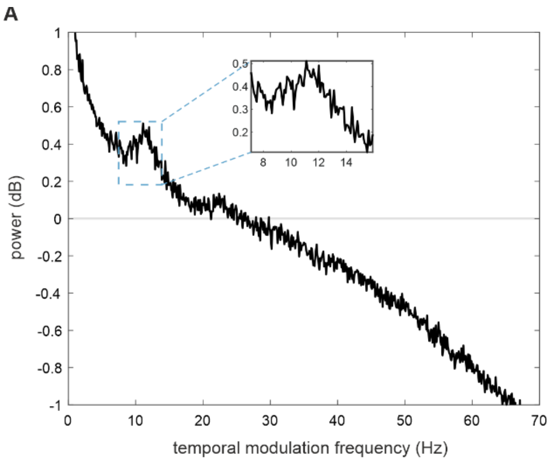

Fig. S1.

Temporal modulation rate of spontaneous vocalizations in C. perspicillata colony is ~ 11 Hz. (**A)** Audio recordings made in our colony of *Carollia* bats (160 minutes total) were highpass filtered (10 kHz) before amplitude envelopes were extracted via the secant method (interpolation over every 5,000 points). The power spectrum density (PSD) of the envelope was computed for each audio segment (10 s each) and averaged across segments. A "control" PSD was computed by randomly permuting sections of the amplitude envelopes in each segment and averaging the permuted-segment PSDs. The "control" PSD was subtracted from original average PSD to produce a power spectrum normalized for ambient noise/power in the signal. The peak of this power spectrum was at ~ 11 Hz (inset), indicating the temporal modulation rate of spontaneous calling in the colony.

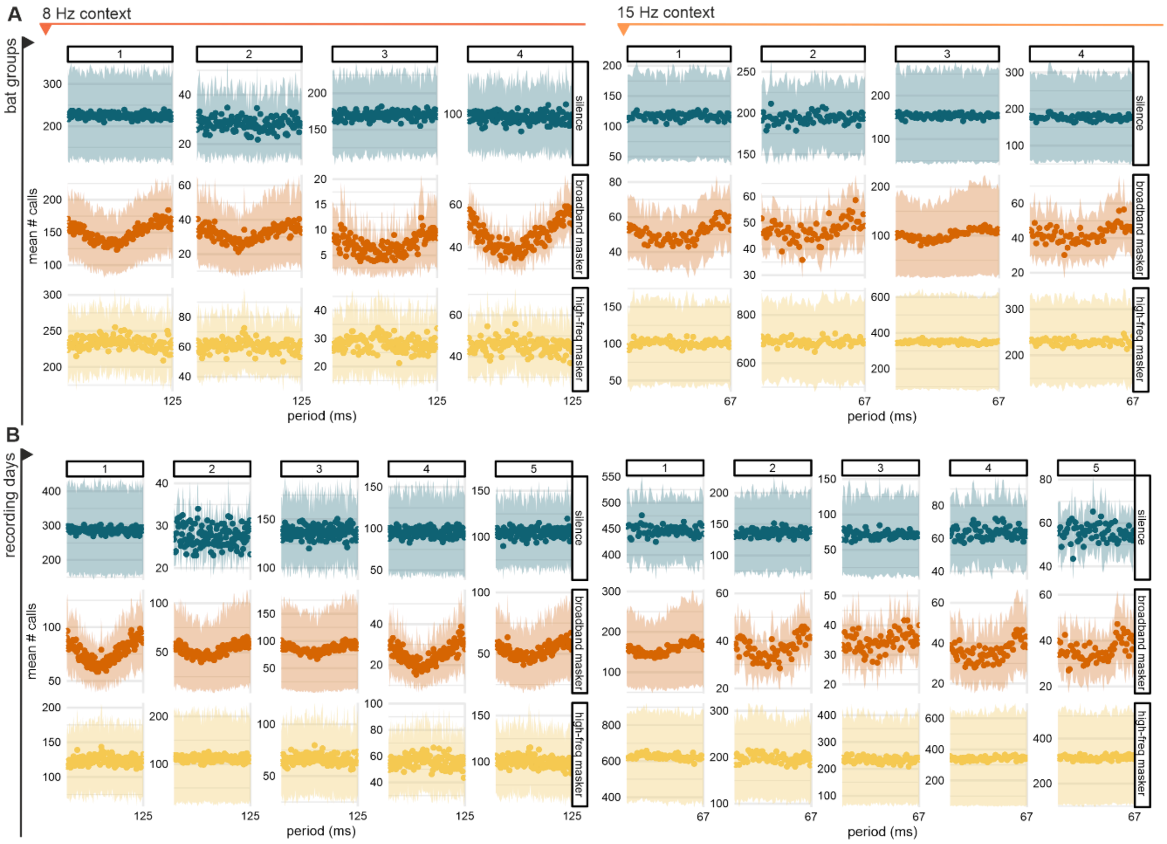

Fig. S2.

Experiment 1: Anti-phase calling is present in all groups and all recording days.

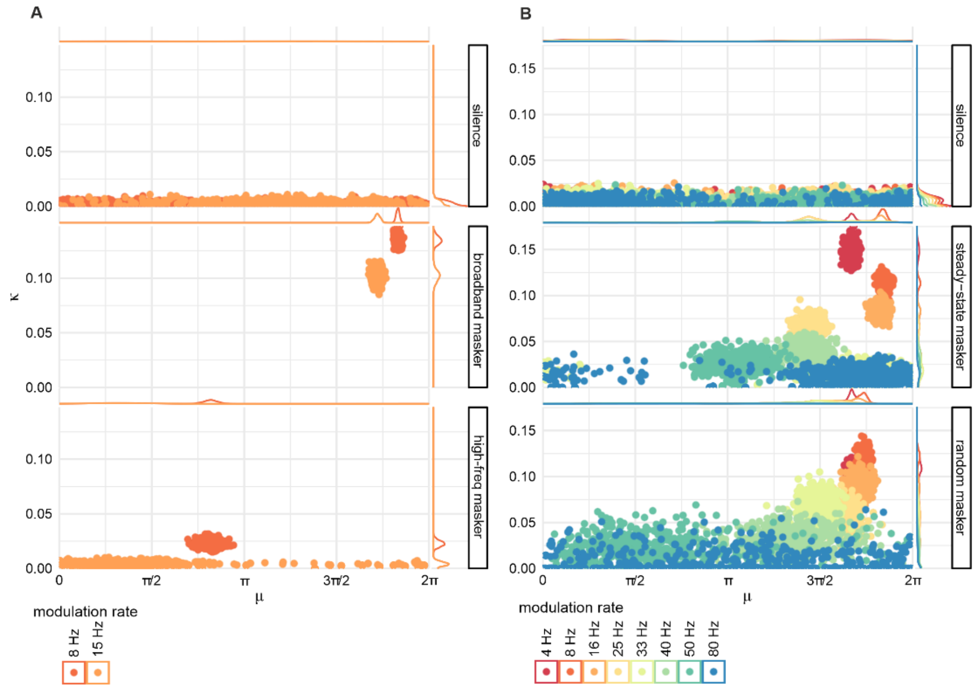

Fig. S3.

Bootstrapped von Mises parameters mean (μ) and concentration (κ) in the cartesian plane. (A) Parameters from experiment 1 data. **(B)** Parameters from experiment 2 data.

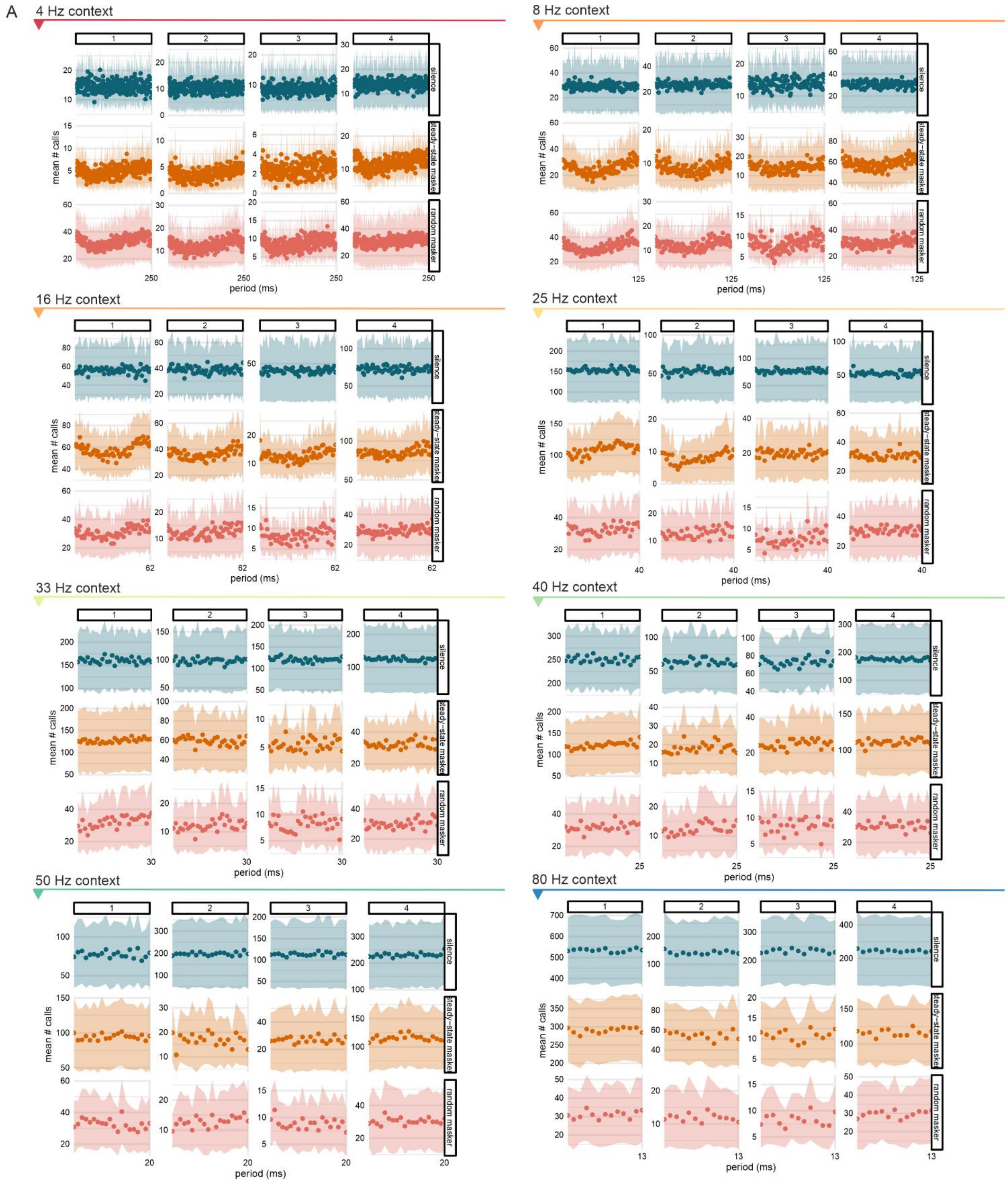

Fig. S4.

Experiment 2: Anti-phase calling is present in all groups for modulation rate conditions 4, 8 and 16. 25 Hz context reveals a slight modulation in calling pattern for some groups between the two masking conditions.

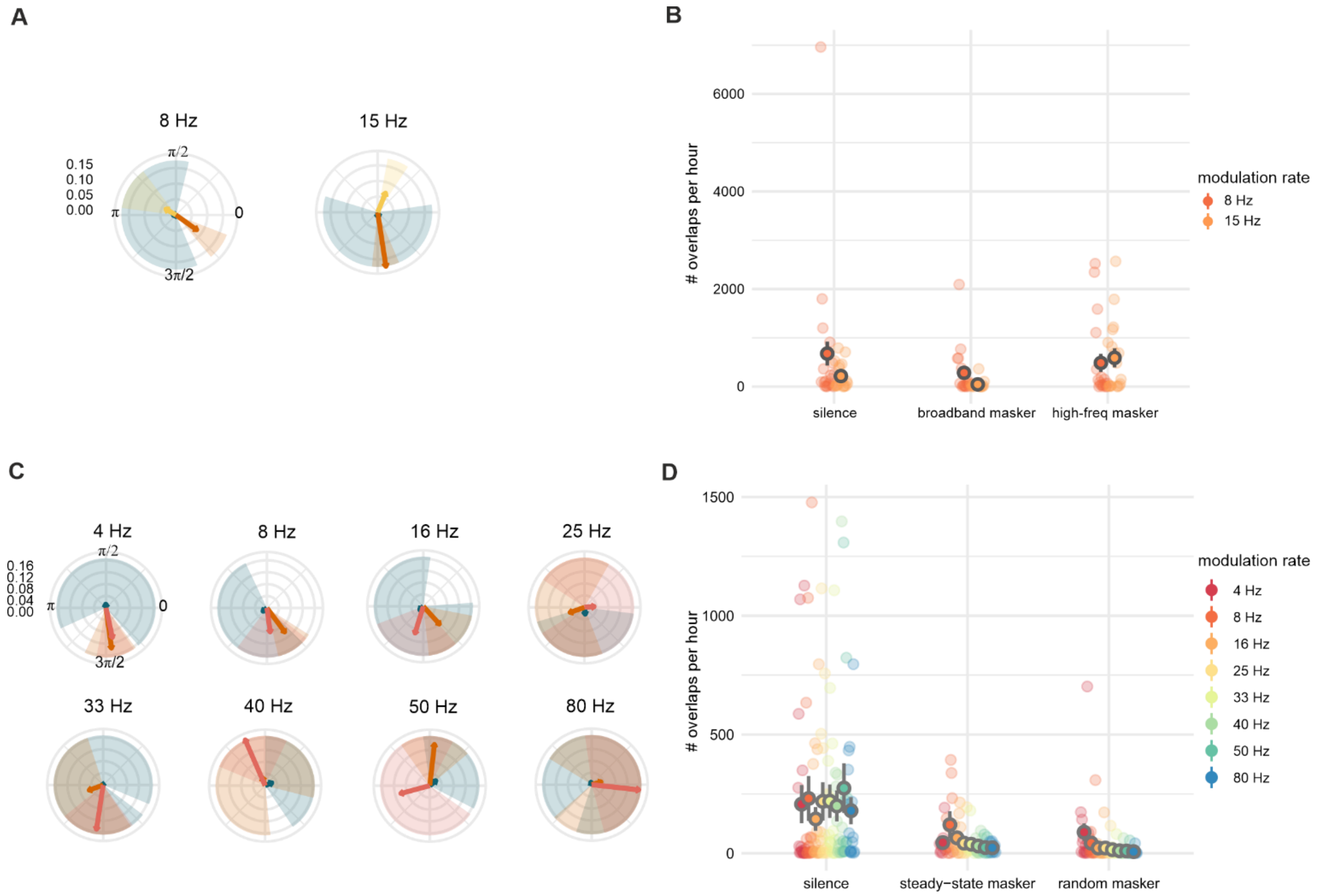

Fig. S5.

Temporal overlap between calls is reduced in the presence of noise. (A) Angular vectors indicate angular mean and concentration for overlapping calls. Shaded segments indicate 95% confidence intervals for angular means. Significant clustering is seen in the 8Hz broadband and 15Hz high-freq masking condition. **(B)** For experiment 1, estimated mean number of overlapping calls (darkly colored dots with gray outline) observed per hour are reduced in broadband masking noise compared to baseline. Lightly colored dots indicate number of overlaps observed per hour for each group for each recording day. Gray vertical lines indicate SE of model fit for predicted means. **(C)** For experiment 2, overlapping calls are significantly clustered only at 8 and 16Hz in the steady-state condition. (**D)** For experiment 2, estimated mean overlapping calls (darkly colored dots with gray outline) dropped significantly between silent baseline and masking conditions for all modulation rates except 4 Hz. Lightly colored dots indicate number of overlaps observed per hour for each group for each recording day. Gray vertical lines indicate SE of model fit for predicted means.

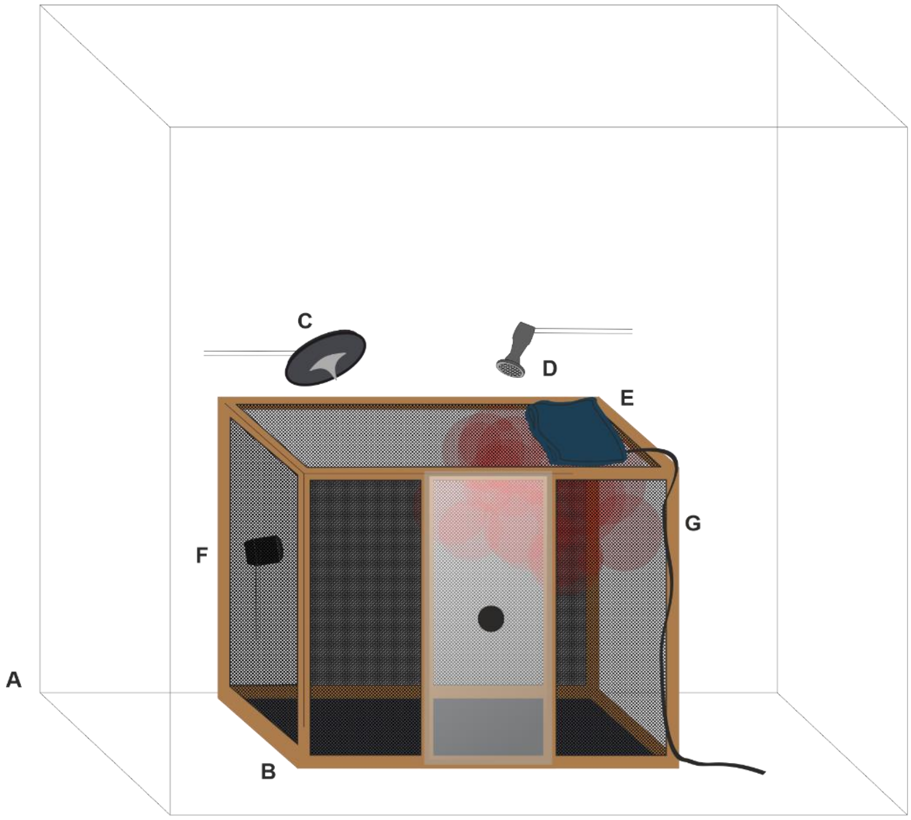

Fig. S6.

Schematic of experimental setup. (a) Recordings were performed inside a ~120 x 112 x 78 cm (w x h x d, interior measurements), sound-isolated chamber. Bats were placed inside an inner box **(b)** measuring 60 x 47 x 30 cm, where they could freely flit and socialize. A directional speaker **(c)** and Avisoft microphone **(d)** were placed outside the box. A heating pad **(e)** provided warmth and resulted in the bats consistently roosting in the upper right corner of the box. Pink circles **(g)** provide a coarse heatmap (estimated from visual inspection) of the locations where the bats roosted the most. Video was recorded using a webcam placed inside the inner box **(f)** and used to confirm the location of the bats inside the box. Bats most frequently rooted ~30 cm from the speaker and ~15 cm from the microphone. The speaker and microphone were placed approximately 8 cm from each other and 10 cm above the top of the inner box.

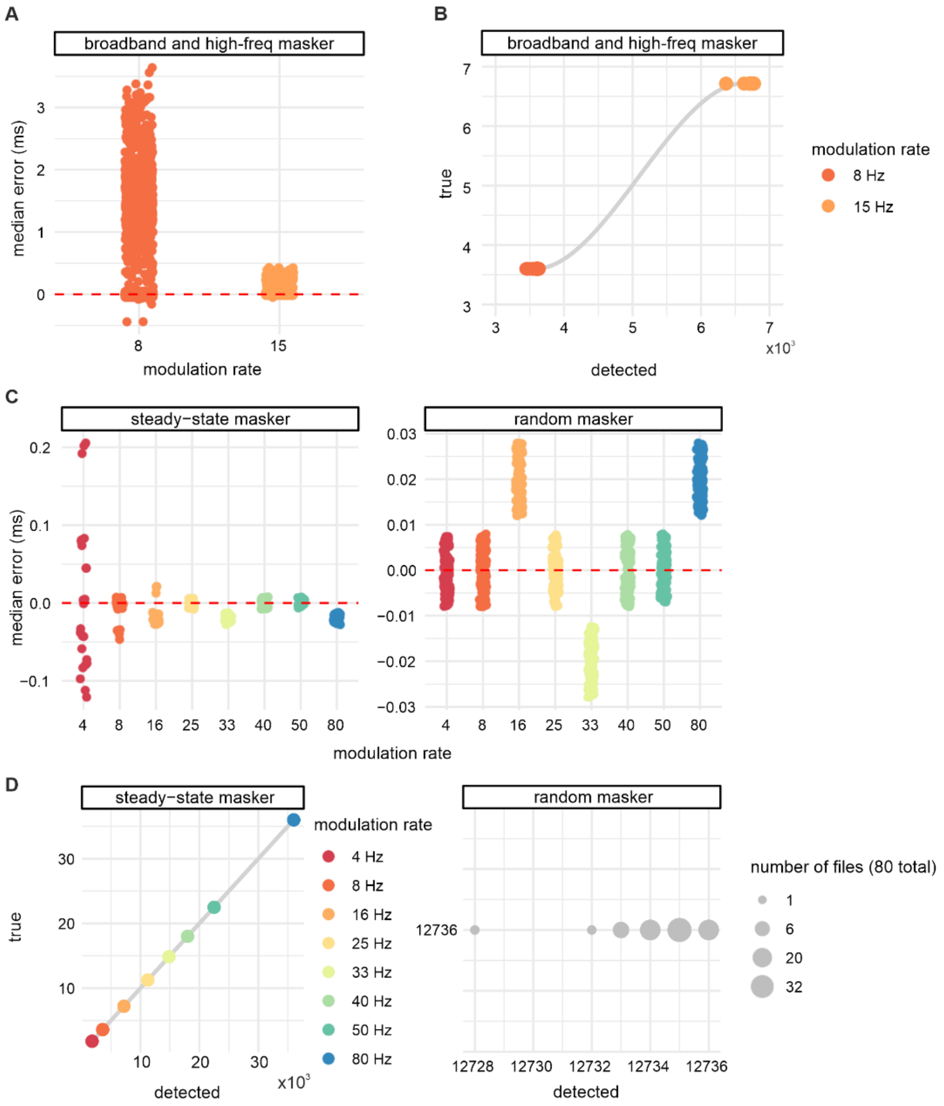

Fig. S7.

Temporal and cycle-wise accuracy of instantaneous phase models. (A) For experiment 1, the median deviation error between expected modulation periods (1/*f*) and derived periods in the instantaneous phase model for all cycles across the entire data set. Errors were mostly between 0 and 3 ms for both modulation rate contexts. **(B)** For experiment 1, the number of true cycles in each one-hour recording block vs. the number of detected cycles in the instantaneous phase model. On average, 5 more cycles were detected in recording blocks in the 8Hz context, and 19 fewer cycles were detected in the 15Hz contexts. **(C)** For experiment 2, median deviation error between expected and derived periods for each modulation rate were less than ±0.3 ms for all rates in both masking conditions. **(D)** For experiment 2, true vs. detected number of cycles were almost perfectly matched for the steady-state condition. On average, detected cycles were ± 2 cycles from expected values (estimated per 7.5 minute block). For the random condition, true vs. detected cycles were calculated irrespective of modulation rate, but were accurate to 0:-4 cycles per 15 minute block (file).

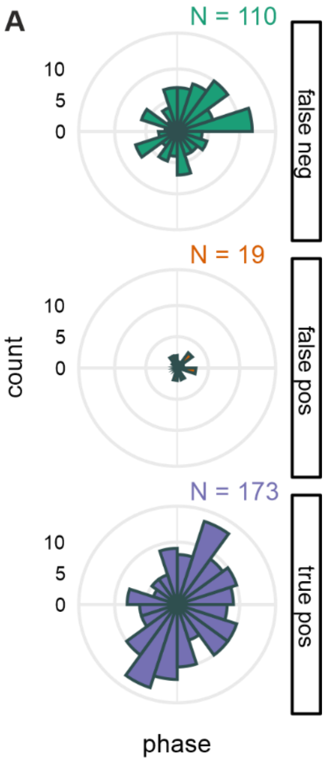

Fig. S8.

DAS-predicted call events by class, as a function of call onset phase. Phase tagging of detected call events from a representative test set (experiment 2: random masker) showed that DAS is not strongly biased to detect calls at particular phases in the amplitude modulation cycle.

Supplementary Tables

Table S1.

Estimated proportion of echolocation to communication calls. A total of 2,673 calls were classified as echolocation or communication calls by visual inspection of spectrograms from all four masking conditions used in both experiments (spectrogram segments sampled from randomly selected groups and files). A weighed average of the proportion of echolocation pulses to communication calls (weighted by the total duration of audio data inspected for each condition) was then computed.

	Playback Condition			
	**Silence**	**Broadband masker/Steady-state masker**	**High-freq masker**	**Random masker**
Total duration scanned (s)	180	240	90	90
Condition-wise mean prop. echo	0.79	0.96	0.94	0.78
Weighted mean prop. echo		0.88		

Table S2.

Experiment 1: Circular statistics for call onsets with respect to the amplitude modulation cycle

		Circular Statistics											
		**Summary Statistics**					**Rayleigh's Test**			**MLE von Mises**			
**modulation**	**condition**	**n**	$\bar{\theta}$	$\bar{r}$	**Vm**	**sd**	**R**	p	$P_{Bonferroni}$	μ_{MLE}	**CI_{lo}**	**CI_{hi}**	κ_{MLE}
8Hz	silence	325,333	0.738	0.001	0.999	3.722	0.001	0.279	1	0.738	4.534	4.327	0.002
8Hz	broadband masker	146,187	5.752	0.067	0.933	2.324	0.058	0.000	<0.001	5.752	5.698	5.808	0.135
8Hz	high-freq masker	229,311	2.579	0.012	0.989	2.989	-0.010	1.000	1	2.579	2.295	2.836	0.023
15Hz	silence	202,822	3.933	0.001	0.999	3.661	-0.001	0.709	1	3.933	1.962	0.506	0.002
15Hz	broadband masker	78,197	5.403	0.051	0.949	2.442	0.032	0.000	<0.001	5.403	5.306	5.504	0.102
15Hz	high-freq masker	443,217	1.042	0.002	0.998	3.524	0.001	0.170	1	1.042	6.003	2.560	0.004

N = number of observations. $\bar{\theta}$ = Mean resultant direction. $\bar{r}$ = Mean resultant length. Vm = circular variance. sd = circular standard deviation. R = test statistics for Rayleigh's test of uniformity. p = p value. $P_{Bonferroni}$ = Bonferroni corrected p-values for the family of all tests in the table. μ_{MLE} = Mean parameter estimated from the maximum likelihood von Mises distribution. CI_{lo}, CI_{hi} = Boostrapped upper and lower thresholds for the 95% confidence interval for the MLE mean parameter. κ_{MLE} = Concentration parameter estimated from the MLE von Mises distribution.

Table S3.

Experiment 1: Rao's test for differences in angular means or dispersions in distributions of call onsets between playback conditions

Rao test for homogeneity of angular means & dispersions within mod rates						
**modulation**	**condition**	**test**	**statistic**	**df**	***p***	**$P_{Bonferroni}$**
8Hz	Omnibus	polar vectors	0.477	2	0.788	1.000
8Hz	Omnibus	dispersions	346.680	2	0.000	<0.001
15Hz	Omnibus	polar vectors	2.708	2	0.258	0.516
15Hz	Omnibus	dispersions	100.345	2	0.000	<0.001
8Hz	silence vs. Broadband masker	polar vectors	0.422	1	0.516	1.00
8Hz	silence vs. Broadband masker	dispersions	332.013	1	0.000	<0.001
8Hz	silence vs. High-freq masker	polar vectors	0.444	1	0.505	1.00
8Hz	silence vs. High-freq masker	dispersions	14.766	1	0.000	<0.001
8Hz	broadband masker vs. High-freq masker	polar vectors	0.054	1	0.817	1.00
8Hz	broadband masker vs. High-freq masker	dispersions	307.346	1	0.000	<0.001
15Hz	silence vs. Broadband masker	polar vectors	0.739	1	0.390	0.78
15Hz	silence vs. Broadband masker	dispersions	100.231	1	0.000	<0.001
15Hz	silence vs. High-freq masker	polar vectors	0.045	1	0.832	1.00
15Hz	silence vs. High-freq masker	dispersions	0.193	1	0.660	1.00
15Hz	broadband masker vs. High-freq masker	polar vectors	1.975	1	0.160	0.32
15Hz	broadband masker vs. High-freq masker	dispersions	100.029	1	0.000	<0.001

Table S4.

Experiment 1: Negative binomial model results for calls emitted both modulation rate contexts

Observed calls per hour in 8Hz modulated noise			
**Predictors**	**Incidence Rate Ratios**	**CI**	***p***
(Intercept)	16266.65	10048.04 – 28874.24	<0.001
condition [broadband masker]	0.45	0.21 – 0.95	0.034
condition [high-freq masker]	0.70	0.33 – 1.49	0.355
Observations	60		
R^2 Nagelkerke	0.097		
Deviance	72.505		
AIC	1239.835		

Observed calls per hour in 15Hz modulated noise			
**Predictors**	**Incidence Rate Ratios**	**CI**	***p***
(Intercept)	10674.84	6729.32 – 18413.99	<0.001
condition [broadband masker]	0.39	0.19 – 0.79	0.008
condition [high-freq masker]	2.19	1.07 – 4.46	0.030
Observations	57		
R^2 Nagelkerke	0.395		
Deviance	67.547		
AIC	1171.104		

Note that the p-values in the table above are not Bonferroni corrected, as they represent p-values for model coefficients.

Table S5.

Experiment 1: Incidence rate ratios for calling derived from negative binomial models

Incidence Rate Ratios				
**modulation**	**coefficient**	**Estimate**	**2.5 %**	**97.5 %**
8Hz	(Intercept)	16266.65	10048.04	28874.24
8Hz	broadband masker	0.45	0.21	0.95
8Hz	high-freq masker	0.70	0.33	1.49
15Hz	(Intercept)	10674.84	6729.32	18413.99
15Hz	broadband masker	0.39	0.19	0.79
15Hz	high-freq masker	2.19	1.07	4.46

Table S6.

Experiment 1: ANOVA Analysis of variance for the predictor of playback condition for each modulation rate model, separately.

		Type II Analysis of Deviance		
**model**	**predictor**	**LR Chi-square**	**df**	***p***
8Hz	condition	4.37	2	0.11
15Hz	condition	21.34	2	<0.001

Table S7.

Experiment 1: Estimated marginal means of call incidence rates per playback condition

Estimated Marginal Means							
**modulation**	**contrast**	**IRR**	**se**	**CI_{lo}**	**CI_{hi}**	**Z ratio**	***p***
8Hz	silence / (broadband masker)	2.22	0.84	0.90	5.50	2.12	0.10
8Hz	silence / (high-freq masker)	1.42	0.54	0.57	3.51	0.92	1.00
8Hz	(broadband masker) / (high-freq masker)	0.64	0.24	0.26	1.58	-1.19	0.70
15Hz	silence / (broadband masker)	2.59	0.94	1.09	6.15	2.64	0.03
15Hz	silence / (high-freq masker)	0.46	0.16	0.19	1.08	-2.17	0.09
15Hz	(broadband masker) / (high-freq masker)	0.18	0.06	0.07	0.42	-4.81	<0.001

IRR = Incidence rate ratios.

Table S8.

Experiment 2: Circular statistics for call onsets with respect to the amplitude modulation cycle

		Circular Statistics											
		**Summary Statistics**				**Rayleigh's Test**				**MLE von Mises**			
**modulation**	**condition**	**n**	$\bar{\theta}$	$\bar{r}$	**Vm**	**sd**	**R**	p	$p_{Bonferroni}$	μ_{MLE}	CI_{lo}	CI_{hi}	κ_{MLE}
4Hz	silence	59,635	6.203	0.003	0.997	3.388	0.003	0.134	1	6.203	4.212	3.124	0.006
4Hz	steady-state masker	28,938	5.246	0.074	0.926	2.282	0.038	0.000	<0.001	5.246	5.140	5.358	0.148
4Hz	random masker	105,990	5.245	0.054	0.946	2.416	0.027	0.000	<0.001	5.245	5.170	5.319	0.108
8Hz	silence	62,203	5.480	0.002	0.998	3.462	0.002	0.270	1	5.480	3.523	2.293	0.005
8Hz	steady-state masker	65,446	5.795	0.057	0.943	2.395	0.050	0.000	<0.001	5.795	5.702	5.891	0.114
8Hz	random masker	51,939	5.459	0.061	0.939	2.366	0.041	0.000	<0.001	5.459	5.356	5.554	0.122
16Hz	silence	65,769	0.858	0.003	0.997	3.376	0.002	0.213	1	0.858	5.075	3.812	0.007
16Hz	steady-state masker	60,482	5.732	0.042	0.958	2.520	0.036	0.000	<0.001	5.732	5.603	5.871	0.084
16Hz	random masker	25,543	5.376	0.046	0.955	2.486	0.028	0.000	<0.001	5.376	5.178	5.575	0.091
25Hz	silence	67,403	4.819	0.003	0.997	3.373	0.000	0.448	1	4.819	2.541	0.274	0.007
25Hz	steady-state masker	32,743	4.535	0.032	0.968	2.618	-0.006	0.928	1	4.535	4.299	4.766	0.065
25Hz	random masker	16,600	5.056	0.027	0.973	2.689	0.009	0.050	1	5.056	4.653	5.478	0.054
33Hz	silence	78,179	0.895	0.004	0.996	3.313	0.003	0.153	1	0.895	5.646	2.632	0.008
33Hz	steady-state masker	34,843	5.359	0.008	0.992	3.093	0.005	0.092	1	5.359	4.466	0.165	0.017
33Hz	random masker	12,830	4.688	0.034	0.966	2.599	-0.001	0.552	1	4.688	4.358	5.069	0.068
40Hz	silence	68,797	0.196	0.001	0.999	3.668	0.001	0.331	1	0.196	4.283	2.335	0.002
40Hz	steady-state masker	34,176	4.486	0.020	0.980	2.801	-0.004	0.877	1	4.486	4.063	4.874	0.040
40Hz	random masker	10,531	4.241	0.015	0.985	2.891	-0.007	0.844	1	4.241	3.404	5.347	0.031
50Hz	silence	61,956	5.052	0.002	0.998	3.564	0.001	0.419	1	5.052	2.940	1.454	0.004
50Hz	steady-state masker	25,425	3.302	0.013	0.987	2.937	-0.013	0.999	1	3.302	2.584	4.017	0.027
50Hz	random masker	8,487	1.776	0.009	0.991	3.055	-0.002	0.599	1	1.776	5.797	3.788	0.019
80Hz	silence	75,144	0.679	0.002	0.998	3.457	0.002	0.221	1	0.679	4.565	3.322	0.005
80Hz	steady-state masker	30,675	5.427	0.007	0.993	3.166	0.004	0.139	1	5.427	3.897	0.352	0.013
80Hz	random masker	5,260	4.324	0.002	0.998	3.561	-0.001	0.527	1	4.324	1.952	2.322	0.004

Table S9.

Experiment 2: Watson-Wheeler tests for the circular homogeneity of distributions of call onsets

Watson-Wheeler test for homogeneity of groups (within modulation rates)			
**modulation**	**statistic**	***p***	$\boldsymbol{p_{Bonferroni}}$
4Hz	281.693	<0.001	<0.001
8Hz	273.637	<0.001	<0.001
16Hz	143.040	<0.001	<0.001
25Hz	45.416	<0.001	<0.001
33Hz	32.695	<0.001	<0.001
40Hz	20.606	<0.001	<0.001
50Hz	8.775	0.067	0.536
80Hz	2.286	0.683	1.000

Table S10.

Experiment 2: Rao's test for differences in angular means or dispersions between playback conditions
Omnibus tests were carried out for each modulation rate to test if differences existed between the three playback conditions. For all tests with significant differences in either polar vectors or polar dispersions, post-hoc tests were computed on pairs of playback conditions.

Rao test for homogeneity of angular means & dispersions

**modulation**	**condition**	**test**	**statistic**	**df**	***p***	***p*$_{Bonferroni}$**
4Hz	Omnibus	polar vectors	3.110	2	0.211	0.422
4Hz	Omnibus	dispersions	232.370	2	<0.001	<0.001
8Hz	Omnibus	polar vectors	18.883	2	<0.001	<0.001
8Hz	Omnibus	dispersions	199.932	2	<0.001	<0.001
16Hz	Omnibus	polar vectors	6.895	2	0.032	0.064
16Hz	Omnibus	dispersions	77.400	2	<0.001	<0.001
25Hz	Omnibus	polar vectors	3.853	2	0.146	0.292
25Hz	Omnibus	dispersions	22.588	2	<0.001	<0.001
33Hz	Omnibus	polar vectors	1.668	2	0.434	0.868
33Hz	Omnibus	dispersions	7.870	2	0.020	0.040
40Hz	Omnibus	polar vectors	0.897	2	0.639	1.000
40Hz	Omnibus	dispersions	7.848	2	0.020	0.040
50Hz	Omnibus	polar vectors	0.106	2	0.948	1.000
50Hz	Omnibus	dispersions	2.521	2	0.284	0.568
80Hz	Omnibus	polar vectors	0.806	2	0.668	1.000
80Hz	Omnibus	dispersions	0.492	2	0.782	1.000
4Hz	silence vs. steady-state masker	polar vectors	2.985	1	0.084	0.168
4Hz	silence vs. steady-state masker	dispersions	79.850	1	0.000	0.000
4Hz	silence vs. random masker	polar vectors	3.087	1	0.079	0.158
4Hz	silence vs. random masker	dispersions	153.051	1	0.000	0.000
4Hz	steady-state masker vs. random masker	polar vectors	0.000	1	0.983	1.000
4Hz	steady-state masker vs. random masker	dispersions	15.231	1	0.000	0.000
8Hz	silence vs. steady-state masker	polar vectors	0.046	1	0.830	1.000
8Hz	silence vs. steady-state masker	dispersions	104.750	1	0.000	0.000
8Hz	silence vs. random masker	polar vectors	0.000	1	0.985	1.000
8Hz	silence vs. random masker	dispersions	95.520	1	0.000	0.000
8Hz	steady-state masker vs. random masker	polar vectors	18.858	1	0.000	0.000
8Hz	steady-state masker vs. random masker	dispersions	0.944	1	0.331	0.662
8Hz	silence vs. steady-state masker	polar vectors	0.046	1	0.830	1.000
8Hz	silence vs. steady-state masker	dispersions	104.750	1	0.000	0.000
8Hz	silence vs. random masker	polar vectors	0.000	1	0.985	1.000
8Hz	silence vs. random masker	dispersions	95.520	1	0.000	0.000
8Hz	steady-state masker vs. random masker	polar vectors	18.858	1	0.000	0.000
8Hz	steady-state masker vs. random masker	dispersions	0.944	1	0.331	0.662

Table S11.

Experiment 2: Estimated marginal means of call incidence rates across modulation rates

Estimated Marginal Means							
**modulation**	**contrast**	**IRR**	**se**	**CI_{lo}**	**CI_{hi}**	**Z ratio**	***p***
silence	4Hz / 8Hz	0.96	0.47	0.20	4.49	-0.09	1.00
silence	4Hz / 16Hz	0.91	0.45	0.19	4.24	-0.20	1.00
silence	4Hz / 25Hz	0.88	0.44	0.19	4.14	-0.25	1.00
silence	4Hz / 33Hz	0.76	0.38	0.16	3.57	-0.55	1.00
silence	4Hz / 40Hz	0.82	0.41	0.17	3.93	-0.39	1.00
silence	4Hz / 50Hz	0.96	0.48	0.21	4.50	-0.08	1.00
silence	4Hz / 80Hz	0.79	0.39	0.17	3.71	-0.47	1.00
silence	8Hz / 16Hz	0.95	0.47	0.20	4.43	-0.11	1.00
silence	8Hz / 25Hz	0.92	0.46	0.20	4.32	-0.16	1.00
silence	8Hz / 33Hz	0.80	0.39	0.17	3.72	-0.46	1.00
silence	8Hz / 40Hz	0.86	0.43	0.18	4.10	-0.30	1.00
silence	8Hz / 50Hz	1.00	0.50	0.22	4.70	0.01	1.00
silence	8Hz / 80Hz	0.83	0.41	0.18	3.87	-0.38	1.00
silence	16Hz / 25Hz	0.98	0.48	0.21	4.57	-0.05	1.00
silence	16Hz / 33Hz	0.84	0.42	0.18	3.94	-0.35	1.00
silence	16Hz / 40Hz	0.91	0.46	0.19	4.34	-0.19	1.00
silence	16Hz / 50Hz	1.06	0.52	0.23	4.97	0.12	1.00
silence	16Hz / 80Hz	0.88	0.43	0.19	4.10	-0.27	1.00
silence	25Hz / 33Hz	0.86	0.43	0.18	4.04	-0.30	1.00
silence	25Hz / 40Hz	0.93	0.47	0.20	4.44	-0.14	1.00
silence	25Hz / 50Hz	1.09	0.54	0.23	5.09	0.17	1.00
silence	25Hz / 80Hz	0.90	0.44	0.19	4.20	-0.22	1.00
silence	33Hz / 40Hz	1.08	0.54	0.23	5.16	0.15	1.00
silence	33Hz / 50Hz	1.26	0.62	0.27	5.90	0.47	1.00
silence	33Hz / 80Hz	1.04	0.51	0.22	4.87	0.08	1.00
silence	40Hz / 50Hz	1.17	0.58	0.24	5.58	0.31	1.00
silence	40Hz / 80Hz	0.96	0.48	0.20	4.60	-0.07	1.00
silence	50Hz / 80Hz	0.82	0.41	0.18	3.86	-0.39	1.00
steady-state masker	4Hz / 8Hz	0.42	0.21	0.09	2.05	-1.71	1.00
steady-state masker	4Hz / 16Hz	0.48	0.24	0.10	2.29	-1.47	1.00
steady-state masker	4Hz / 25Hz	0.84	0.43	0.17	4.10	-0.34	1.00
steady-state masker	4Hz / 33Hz	0.83	0.42	0.17	3.97	-0.37	1.00
steady-state masker	4Hz / 40Hz	0.80	0.41	0.16	3.93	-0.43	1.00
steady-state masker	4Hz / 50Hz	1.14	0.57	0.24	5.44	0.26	1.00
steady-state masker	4Hz / 80Hz	0.94	0.47	0.20	4.51	-0.12	1.00
steady-state masker	8Hz / 16Hz	1.14	0.58	0.23	5.56	0.26	1.00
steady-state masker	8Hz / 25Hz	2.00	1.03	0.40	9.95	1.35	1.00
steady-state masker	8Hz / 33Hz	1.98	1.00	0.41	9.65	1.34	1.00
steady-state masker	8Hz / 40Hz	1.92	0.98	0.38	9.54	1.26	1.00
steady-state masker	8Hz / 50Hz	2.71	1.38	0.56	13.22	1.96	1.00
steady-state masker	8Hz / 80Hz	2.25	1.14	0.46	10.96	1.59	1.00
steady-state masker	16Hz / 25Hz	1.75	0.89	0.36	8.56	1.11	1.00
steady-state masker	16Hz / 33Hz	1.74	0.87	0.36	8.30	1.10	1.00
steady-state masker	16Hz / 40Hz	1.68	0.85	0.34	8.20	1.02	1.00
steady-state masker	16Hz / 50Hz	2.38	1.19	0.50	11.37	1.73	1.00
steady-state masker	16Hz / 80Hz	1.97	0.99	0.41	9.43	1.35	1.00
steady-state masker	25Hz / 33Hz	0.99	0.50	0.20	4.83	-0.02	1.00
steady-state masker	25Hz / 40Hz	0.96	0.49	0.19	4.77	-0.08	1.00
steady-state masker	25Hz / 50Hz	1.36	0.69	0.28	6.62	0.60	1.00
steady-state masker	25Hz / 80Hz	1.12	0.57	0.23	5.48	0.23	1.00
steady-state masker	33Hz / 40Hz	0.97	0.49	0.20	4.73	-0.06	1.00

Estimated Marginal Means

**modulation**	**contrast**	**IRR**	**se**	**CI_{lo}**	**CI_{hi}**	**Z ratio**	***p***
steady-state masker	33Hz / 50Hz	1.37	0.69	0.29	6.55	0.63	1.00
steady-state masker	33Hz / 80Hz	1.14	0.57	0.24	5.43	0.25	1.00
steady-state masker	40Hz / 50Hz	1.42	0.72	0.29	6.91	0.68	1.00
steady-state masker	40Hz / 80Hz	1.17	0.60	0.24	5.72	0.31	1.00
steady-state masker	50Hz / 80Hz	0.83	0.42	0.17	3.96	-0.38	1.00
random masker	4Hz / 8Hz	2.04	0.88	0.53	7.82	1.66	1.00
random masker	4Hz / 16Hz	4.15	1.78	1.08	15.89	3.31	0.03
random masker	4Hz / 25Hz	6.38	2.74	1.67	24.46	4.31	<0.001
random masker	4Hz / 33Hz	8.26	3.55	2.16	31.65	4.91	<0.001
random masker	4Hz / 40Hz	10.06	4.33	2.63	38.56	5.37	<0.001
random masker	4Hz / 50Hz	12.49	5.37	3.26	47.85	5.87	<0.001
random masker	4Hz / 80Hz	20.15	8.67	5.26	77.22	6.98	<0.001
random masker	8Hz / 16Hz	2.03	0.87	0.53	7.79	1.65	1.00
random masker	8Hz / 25Hz	3.13	1.34	0.82	11.98	2.65	0.22
random masker	8Hz / 33Hz	4.05	1.74	1.06	15.51	3.25	0.03
random masker	8Hz / 40Hz	4.93	2.12	1.29	18.90	3.71	0.01
random masker	8Hz / 50Hz	6.12	2.63	1.60	23.45	4.21	<0.001
random masker	8Hz / 80Hz	9.87	4.25	2.58	37.84	5.32	<0.001
random masker	16Hz / 25Hz	1.54	0.66	0.40	5.89	1.00	1.00
random masker	16Hz / 33Hz	1.99	0.86	0.52	7.63	1.60	1.00
random masker	16Hz / 40Hz	2.43	1.04	0.63	9.29	2.06	1.00
random masker	16Hz / 50Hz	3.01	1.29	0.78	11.53	2.56	0.29
random masker	16Hz / 80Hz	4.86	2.09	1.27	18.61	3.67	0.01
random masker	25Hz / 33Hz	1.29	0.56	0.34	4.96	0.60	1.00
random masker	25Hz / 40Hz	1.58	0.68	0.41	6.04	1.06	1.00
random masker	25Hz / 50Hz	1.96	0.84	0.51	7.50	1.56	1.00
random masker	25Hz / 80Hz	3.16	1.36	0.82	12.10	2.67	0.21
random masker	33Hz / 40Hz	1.22	0.52	0.32	4.67	0.46	1.00
random masker	33Hz / 50Hz	1.51	0.65	0.39	5.79	0.96	1.00
random masker	33Hz / 80Hz	2.44	1.05	0.64	9.35	2.07	1.00
random masker	40Hz / 50Hz	1.24	0.53	0.32	4.76	0.50	1.00
random masker	40Hz / 80Hz	2.00	0.86	0.52	7.68	1.61	1.00
random masker	50Hz / 80Hz	1.61	0.69	0.42	6.19	1.11	1.00

Table S12.

Experiment 2: Estimated marginal means for incidence rates of calling

		Estimated Marginal Means					
**modulation**	**contrast**	**IRR**	**se**	**CI_{lo}**	**CI_{hi}**	**Z ratio**	***p***
4Hz	silence / (steady-state masker)	2.06	0.95	0.69	6.20	1.57	0.35
4Hz	silence / random masker	0.56	0.26	0.19	1.69	-1.25	0.63
4Hz	(steady-state masker) / random masker	0.27	0.13	0.09	0.82	-2.82	0.01
8Hz	silence / (steady-state masker)	0.90	0.47	0.26	3.10	-0.20	1.00
8Hz	silence / random masker	1.20	0.61	0.36	4.04	0.36	1.00
8Hz	(steady-state masker) / random masker	1.33	0.68	0.39	4.55	0.55	1.00
16Hz	silence / (steady-state masker)	1.09	0.48	0.38	3.10	0.19	1.00
16Hz	silence / random masker	2.58	1.13	0.90	7.34	2.16	0.09
16Hz	(steady-state masker) / random masker	2.37	1.04	0.83	6.75	1.97	0.15
25Hz	silence / (steady-state masker)	1.96	0.99	0.58	6.54	1.33	0.55
25Hz	silence / random masker	4.06	2.02	1.23	13.36	2.82	0.01
25Hz	(steady-state masker) / random masker	2.08	1.05	0.62	6.94	1.45	0.44
33Hz	silence / (steady-state masker)	2.24	1.09	0.70	7.16	1.67	0.29
33Hz	silence / random masker	6.09	2.95	1.91	19.44	3.73	<0.001
33Hz	(steady-state masker) / random masker	2.72	1.32	0.85	8.66	2.06	0.12
40Hz	silence / (steady-state masker)	2.01	0.95	0.65	6.26	1.48	0.42
40Hz	silence / random masker	6.88	3.22	2.24	21.08	4.12	<0.001
40Hz	(steady-state masker) / random masker	3.42	1.60	1.11	10.47	2.62	0.03
50Hz	silence / (steady-state masker)	2.44	1.22	0.74	8.04	1.79	0.22
50Hz	silence / random masker	7.30	3.64	2.21	24.08	3.99	<0.001
50Hz	(steady-state masker) / random masker	3.00	1.49	0.91	9.88	2.20	0.08
80Hz	silence / (steady-state masker)	2.45	1.11	0.82	7.28	1.97	0.15
80Hz	silence / random masker	14.29	6.50	4.80	42.50	5.84	<0.001
80Hz	(steady-state masker) / random masker	5.83	2.66	1.96	17.35	3.87	<0.001

Table S13.

Experiment 2: Incidence rate ratios derived from negative binomial models

		Incidence Rate Ratios		
**modulation**	**coefficient**	**Estimate**	**2.5 %**	**97.5 %**
4Hz	(Intercept)	2981.75	1675.19	6085.74
4Hz	steady-state masker	0.49	0.19	1.21
4Hz	random masker	1.78	0.71	4.45
8Hz	(Intercept)	3110.15	1655.22	6909.31
8Hz	steady-state masker	1.11	0.40	3.12
8Hz	random masker	0.83	0.30	2.31
16Hz	(Intercept)	3288.45	1895.48	6456.38
16Hz	steady-state masker	0.92	0.38	2.20
16Hz	random masker	0.39	0.16	0.93
25Hz	(Intercept)	3370.15	1815.43	7344.02
25Hz	steady-state masker	0.51	0.19	1.41
25Hz	random masker	0.25	0.09	0.67
33Hz	(Intercept)	3908.95	2136.58	8324.83
33Hz	steady-state masker	0.45	0.17	1.17
33Hz	random masker	0.16	0.06	0.43
40Hz	(Intercept)	3620.89	2003.11	7568.02
40Hz	steady-state masker	0.50	0.19	1.28
40Hz	random masker	0.15	0.06	0.37
50Hz	(Intercept)	3097.80	1667.09	6760.91
50Hz	steady-state masker	0.41	0.15	1.11
50Hz	random masker	0.14	0.05	0.37
80Hz	(Intercept)	3757.20	2122.84	7602.55
80Hz	steady-state masker	0.41	0.16	1.01
80Hz	random masker	0.07	0.03	0.17

Table S14.

Experiment 2: ANOVA. Analysis of variance for the predictor of playback condition for each negative binomial model, separately.

Type II Analysis of Deviance				
**model**	**predictor**	**LR Chi-square**	**df**	***p***
4Hz	condition	7.55	2	0.02
8Hz	condition	0.31	2	0.86
16Hz	condition	5.07	2	0.08
25Hz	condition	7.55	2	0.02
33Hz	condition	12.74	2	<0.001
40Hz	condition	15.16	2	<0.001
50Hz	condition	14.36	2	<0.001
80Hz	condition	27.63	2	<0.001

Table S15.

Number of temporally overlapping calls across both experiments in each condition

	Silent	Broadband masker (Steady-state masker)	High-freq masker	Random masker
Experiment 1	17,645	5,627	19,346	-
Experiment 2	24,903	4,093	-	2,946

Table S16.

Estimated marginal means for overlapping calls. Estimated marginal mean differences in call incidence rates calculated on negative binomial models for overlapping calls for each modulation rate, separately.

			Estimated Marginal Means					
**experiment**	**modulation**	**contrast**	**IRR**	**se**	**CI_{lo}**	**CI_{hi}**	**Z ratio**	***p***
1	8Hz	silence / (broadband masker)	2.40	1.28	0.66	8.65	1.63	0.31
	8Hz	silence / (high-freq masker)	1.40	0.74	0.39	4.94	0.63	1.00
	8Hz	(broadband masker) / (high-freq masker)	0.58	0.32	0.16	2.17	-0.98	0.98
	15Hz	silence / (broadband masker)	4.73	2.19	1.56	14.36	3.35	<0.001
	15Hz	silence / (high-freq masker)	0.37	0.17	0.12	1.11	-2.17	0.09
	15Hz	(broadband masker) / (high-freq masker)	0.08	0.04	0.03	0.24	-5.44	<0.001
2	4Hz	silence / (steady-state masker)	4.58	3.04	0.94	22.45	2.29	0.07
	4Hz	silence / random masker	2.33	1.33	0.60	9.11	1.49	0.41
	4Hz	(steady-state masker) / random masker	0.51	0.34	0.10	2.58	-1.00	0.96
	8Hz	silence / (steady-state masker)	1.92	1.21	0.43	8.65	1.04	0.90
	8Hz	silence / random masker	5.36	3.16	1.31	21.96	2.85	0.01
	8Hz	(steady-state masker) / random masker	2.79	1.78	0.60	12.88	1.61	0.32
	16Hz	silence / (steady-state masker)	2.21	1.16	0.63	7.77	1.52	0.39
	16Hz	silence / random masker	6.56	3.32	1.95	22.02	3.72	0.00
	16Hz	(steady-state masker) / random masker	2.96	1.60	0.81	10.83	2.00	0.14
	25Hz	silence / (steady-state masker)	5.14	3.10	1.21	21.80	2.71	0.02
	25Hz	silence / random masker	9.89	5.80	2.43	40.26	3.91	0.00
	25Hz	(steady-state masker) / random masker	1.93	1.27	0.40	9.37	0.99	0.96
	33Hz	silence / (steady-state masker)	5.72	3.07	1.58	20.69	3.24	0.00
	33Hz	silence / random masker	14.70	7.47	4.35	49.66	5.28	0.00
	33Hz	(steady-state masker) / random masker	2.57	1.50	0.64	10.41	1.62	0.32
	40Hz	silence / (steady-state masker)	6.48	3.29	1.92	21.87	3.68	0.00
	40Hz	silence / random masker	18.39	9.20	5.55	60.93	5.82	0.00
	40Hz	(steady-state masker) / random masker	2.84	1.53	0.78	10.35	1.93	0.16
	50Hz	silence / (steady-state masker)	11.27	6.43	2.88	44.16	4.25	0.00
	50Hz	silence / random masker	27.70	15.51	7.26	105.80	5.93	0.00
	50Hz	(steady-state masker) / random masker	2.46	1.45	0.60	10.12	1.52	0.38
	80Hz	silence / (steady-state masker)	7.69	3.88	2.30	25.73	4.04	0.00
	80Hz	silence / random masker	26.34	13.56	7.68	90.35	6.35	0.00
	80Hz	(steady-state masker) / random masker	3.43	1.94	0.89	13.24	2.18	0.09

Table S17.

Call timings relative to acoustic landmarks

Mean Call Onset Timings						
			**time from peaks (ms)**		**time to trough (ms)**	
**experiment**	**modulation**	**condition**	**median**	**iqr**	**median**	**iqr**
**1**	**All**	broadband masker	37.7	27.9	10.1	1
	8Hz	broadband masker	52.0	0.7	10.5	0.7
	15Hz	broadband masker	24.1	0.7	9.6	0.7
**2**	**All**	steady-state masker	9.3	28.7	8.4	5.8
	**All**	random masker	9.6	26.5	9.0	8.4
	4Hz	steady-state masker	83.7	2.8	41.3	2.8
	4Hz	random masker	83.7	2.0	41.3	2.0
	8Hz	steady-state masker	52.8	1.3	9.7	1.3
	8Hz	random masker	46.1	1.4	16.4	1.4
	16Hz	steady-state masker	25.8	0.9	5.0	0.9
	16Hz	random masker	22.2	1.3	8.6	1.3
	25Hz	steady-state masker	8.9	1.0	11.1	1.0
	25Hz	random masker	12.2	1.7	7.8	1.7
	33Hz	steady-state masker	10.5	2.9	4.3	2.9
	33Hz	random masker	7.4	1.2	7.4	1.2
	40Hz	steady-state masker	5.3	1.0	7.2	1.0
	40Hz	random masker	4.4	2.5	8.1	2.5
	50Hz	steady-state masker	0.5	1.4	9.5	1.4
	50Hz	random masker	-4.1	3.8	14.1	3.8
	80Hz	steady-state masker	4.2	1.8	2.5	1.8
	80Hz	random masker	0.5	5.8	6.2	5.8
