# Supplementary material for: Flexible control of vocal timing in Carollia perspicillata bats enables escape from acoustic interference
Source: Commun Biol. 2023 Nov 13;6:1153. doi: 10.1038/s42003-023-05507-5 (PMC10643407; doi:10.1038/s42003-023-05507-5)
Supplement: Supplementary file 2 — Reporting Summary [file 42003_2023_5507_MOESM2_ESM.pdf]

nature portfolio

Corresponding author(s): Ava Kiai
Julio C. Hechavarria

Last updated by author(s): Aug 30, 2023

Reporting Summary

Nature Portfolio wishes to improve the reproducibility of the work that we publish. This form provides structure for consistency and transparency in reporting. For further information on Nature Portfolio policies, see our Editorial Policies and the Editorial Policy Checklist.

Statistics

For all statistical analyses, confirm that the following items are present in the figure legend, table legend, main text, or Methods section.

n/a	Confirmed	
☐	☒	The exact sample size (*n*) for each experimental group/condition, given as a discrete number and unit of measurement
☐	☒	A statement on whether measurements were taken from distinct samples or whether the same sample was measured repeatedly
☐	☒	The statistical test(s) used AND whether they are one- or two-sided<br>*Only common tests should be described solely by name; describe more complex techniques in the Methods section.*
☐	☒	A description of all covariates tested
☐	☒	A description of any assumptions or corrections, such as tests of normality and adjustment for multiple comparisons
☐	☒	A full description of the statistical parameters including central tendency (e.g. means) or other basic estimates (e.g. regression coefficient) AND variation (e.g. standard deviation) or associated estimates of uncertainty (e.g. confidence intervals)
☐	☒	For null hypothesis testing, the test statistic (e.g. *F*, *t*, *r*) with confidence intervals, effect sizes, degrees of freedom and *P* value noted<br>*Give P values as exact values whenever suitable.*
☒	☐	For Bayesian analysis, information on the choice of priors and Markov chain Monte Carlo settings
☐	☒	For hierarchical and complex designs, identification of the appropriate level for tests and full reporting of outcomes
☐	☒	Estimates of effect sizes (e.g. Cohen's *d*, Pearson's *r*), indicating how they were calculated

Our web collection on statistics for biologists contains articles on many of the points above.

Software and code

Policy information about availability of computer code

Data collection	Data (audio recordings) were recorded via a custom-written Matlab script that controlled simultaneous audio recording and audio playback.
Data analysis	Data were preprocessed (call events detected) in Python 3 using DeepAudioSegmenter and analyzed in R and R Studio. Data and code are available on Github via the link.

For manuscripts utilizing custom algorithms or software that are central to the research but not yet described in published literature, software must be made available to editors and reviewers. We strongly encourage code deposition in a community repository (e.g. GitHub). See the Nature Portfolio guidelines for submitting code & software for further information.

Data

Policy information about availability of data

All manuscripts must include a data availability statement. This statement should provide the following information, where applicable:

- Accession codes, unique identifiers, or web links for publicly available datasets
- A description of any restrictions on data availability
- For clinical datasets or third party data, please ensure that the statement adheres to our policy

Data and scripts used for analysis can be found on Github. DOI: https://zenodo.org/record/7908545

Research involving human participants, their data, or biological material

Policy information about studies with human participants or human data. See also policy information about sex, gender (identity/presentation), and sexual orientation and race, ethnicity and racism.

Reporting on sex and gender	n/a
Reporting on race, ethnicity, or other socially relevant groupings	n/a
Population characteristics	n/a
Recruitment	n/a
Ethics oversight	n/a

Note that full information on the approval of the study protocol must also be provided in the manuscript.

Field-specific reporting

Please select the one below that is the best fit for your research. If you are not sure, read the appropriate sections before making your selection.

☒ Life sciences ☐ Behavioural & social sciences ☐ Ecological, evolutionary & environmental sciences

For a reference copy of the document with all sections, see nature.com/documents/nr-reporting-summary-flat.pdf

Life sciences study design

All studies must disclose on these points even when the disclosure is negative.

Sample size	72 animals were used in total (8 groups in experiment 1, 4 groups in experiment 2). Groups in experiment 1 were determined so that each cell in the design matrix (modulation rate x masking order) would have 2 independent datasets. In experiment 2, we opted for 1 dataset per cell (randomization order x masking order), since we had already observed results from experiment 1 to be robust.
Data exclusions	Experiment 1: For groups 1 & 2 in the 8 Hz context, some recording blocks had buffer issues which caused improper logging of data. Audio files for these blocks were visually checked and sections with corrupted data were removed from the corresponding file if the error was minor (i.e. < 1 second long, or < 3 times per file). If errors were more extensive, the file was removed from analysis. Altogether, approx. 15 minutes of data was removed from the raw data for these two groups combined. For all remaining groups, the first 15 hours of recordings were visually checked for buffer issues. As only a few such occurrences were found, we did not proceed with the visual check.
Replication	In experiment 1, we tested 8 groups of bats. In experiment 2, we tested 4 groups. Main effects reported across all groups were also present at the group level and, within groups, at the level of recording sessions. These data show that our effects were robust and replicable across different subpopulations of our bats.
Randomization	Stimulus presentation order was randomized across bat groups for both experiments.
Blinding	n/a

Reporting for specific materials, systems and methods

We require information from authors about some types of materials, experimental systems and methods used in many studies. Here, indicate whether each material, system or method listed is relevant to your study. If you are not sure if a list item applies to your research, read the appropriate section before selecting a response.

Materials & experimental systems

n/a	Involved in the study	
☒	☐	Antibodies
☒	☐	Eukaryotic cell lines
☒	☐	Palaeontology and archaeology
☐	☒	Animals and other organisms
☒	☐	Clinical data
☒	☐	Dual use research of concern
☒	☐	Plants

Methods

n/a	Involved in the study	
☒	☐	ChIP-seq
☒	☐	Flow cytometry
☒	☐	MRI-based neuroimaging

Animals and other research organisms

Policy information about studies involving animals; ARRIVE guidelines recommended for reporting animal research, and Sex and Gender in Research

Laboratory animals	Carollia perspicillata, adults
Wild animals	n/a
Reporting on sex	Individuals of both sexes were used in this study. More males were used than females (2:1 ratio), since previous studies have shown males to be the more vocal species, and we wished to encourage plenty of vocal behavior. Sex was not used as a variable in any analysis.
Field-collected samples	n/a
Ethics oversight	Regierungspräsidium Darmstadt

Note that full information on the approval of the study protocol must also be provided in the manuscript.
